# A scoping review of the roles, challenges, and strategies for enhancing the performance of community health workers in the response against COVID-19 in low- and middle-income countries

**DOI:** 10.1186/s12875-025-02853-7

**Published:** 2025-05-14

**Authors:** Joseph Mumba Zulu, Adam Silumbwe, Margarate Munakampe, Malizgani Paul Chavula, Chama Mulubwa, Nathanael Sirili, Wanga Zulu, Charles Michelo, Moses Tetui

**Affiliations:** 1 Department of Health Promotion and Education, School of Public Health, University of Zambia, PO Box 50110, Lusaka, Zambia; 2 Department of Health Policy and Management, School of Public Health, University of Zambia, PO Box 50110, Lusaka, Zambia; 3 Department of Epidemiology and Global Health, Umeå University, Umeå, 901 87 Sweden; 4 Yakini Health Research Institute, Lusaka, Zambia; 5 Department of Development Studies, School of Public Health and Social Sciences, Muhimbili University of Health, and Allied Sciences, P.O.BOX 65454, Dar es Salaam, Tanzania; 6 Global Health Institute, Nkwazi Research University, Lusaka, Zambia; 7 School of Public Health Sciences, University of Waterloo, Waterloo, ON Canada

**Keywords:** Community health workers, Health system, COVID-19, LMICs

## Abstract

**Background:**

Global concerns regarding effective response strategies to the COVID-19 pandemic arose amid the swift spread of the virus to low- and middle-income country (LMIC) settings. Although LMICs instituted several measures to mitigate spread of the virus in low resource settings, including task shifting certain demand and supply functions to community actors such as community health workers (CHWs), there remains a lack of synthesized evidence on these experiences and lessons. This scoping review sought to synthesize evidence regarding the roles and challenges faced by CHWs during the fight against COVID-19, along with strategies to address these challenges.

**Methodology:**

We systematically searched several major electronic databases including PubMed, HINARI, Cochrane Library (Reviews and Trials), Science Direct and Google Scholar for relevant literature. The search strategy was designed to capture literature published in LMICs on CHWs roles during COVID-19 period spanning 2019–2023. Two researchers were responsible for retrieving these studies, and critically reviewed them in accordance with Arksey and O’Malley scoping review approach. In total, 22 articles were included and analysed using Clarke and Braun thematic analysis in NVivo 12 Pro Software.

**Results:**

Community health workers (CHWs) played a vital role during the COVID-19 pandemic. They engaged in health promotion and education, conducted surveillance and contact tracing, supported quarantine efforts, and maintained essential primary health services. They also facilitated referrals, advocated for clients and communities, and contributed to vaccination planning and coordination, including tracking and follow-up. However, CHWs faced significant challenges, including a lack of supplies, inadequate infection prevention and control measures, and stigma from community members. Additionally, they encountered limited supportive policies, insufficient remuneration and incentives. To enhance CHWs’ performance, regular training on preventive measures is essential. Utilizing digital technology, such as mobile health, can be beneficial. Establishing collaborative groups through messaging platforms and prioritizing access to COVID-19 vaccines are important steps. Additionally, delivering wellness programs and providing quality protective equipment for CHWs are crucial for their effectiveness.

**Conclusion:**

The study found that CHWs are vital actors within the health system during global pandemics like COVID-19. This entails the need for increased support and investment to better integrate CHWs into health systems during such crises, which could ultimately contribute to sustaining the credibility of CHWs programs and foster more inclusive community health systems (CHSs).

**Supplementary Information:**

The online version contains supplementary material available at 10.1186/s12875-025-02853-7.

## Background

Globally, the critical role of community health workers (CHWs) in providing integrated, quality and people-centered primary healthcare is widely recognized [1, 2]. Numerous advances in health have been recorded over the past three decades in low-and-middle-income countries (LMICs) because of the significant contribution of CHWs [3]. Most LMICs consistently rely on CHWs to deliver reproductive, maternal, newborn, child, and adolescent health services, as well as malaria and HIV/AIDS care—particularly in hard-to-reach communities [4].

Contextual factors such as health system weaknesses, including workforce shortages, have made their role even more essential in LMICs [4]. However, the use of CHWs in national health programs is not exclusive to LMICs. For example, Sweden—a high-income country—reports the engagement of CHWs in establishing virtual health rooms for rural communities [5]. Similarly, there are reports of employing CHWs to improve diabetes care in remote areas among Aboriginal populations in Australia [6].

The mutual application of community resources such as CHWs, to improve overall health outcomes has been dubbed as “galvanizing the community health systems (CHS)” [7]. As a result, there have been growing calls to strengthen the conceptual linkages between the formal health system and the CHS. This is reflected in a recent research agenda on CHS [8]. A CHS is “*a network of local players*,* interactions*,* and practices involved in creating and supporting health in communities. It functions in conjunction with established health structures*” [9].

The emphasis on CHSs during the COVID-19 pandemic was particularly important. A Policy Brief released by the World Health Organization (WHO) on April 1, 2020, outlined 16 proposals to strengthen health systems’ response to COVID-19 [10]. The brief emphasized that early experiences from countries with widespread community transmission underscored the urgent need for the unprecedented deployment of community health system actors. One of the key recommendations was to expand capacity to disseminate COVID-19-related information and manage its flow within health systems. Deploying CHWs to support information dissemination was deemed critical in addressing the widespread misinformation that WHO labeled a COVID-19 “infodemic” [11]. As vaccines became available, community actors such as volunteers played key roles in the running of vaccine clinics in Ontario, Canada [12].

A *Lancet* publication called for the development of a large-scale emergency program to train CHWs in responding to the COVID-19 pandemic [13]. Bezbaruah et al. further emphasized that, given COVID-19’s disproportionate impact on the poor and vulnerable, CHWs played a pivotal role in mitigating the pandemic’s effects, particularly in countries with less resilient health systems [14]. Overall, CHSs were vital in the fight against COVID-19, and investing in them will be crucial in responding to future crises [15].

CHWs matter and remain central to community health because they are trusted members of the community who are often the most accessible point of care [16]. According to Ballard et al., investment in CHSs can help achieve critical pandemic control goals. These include protecting healthcare workers, interrupting virus transmission, maintaining and scaling up existing healthcare services, and shielding the most vulnerable from socioeconomic shocks [17].

While the vital role of community health workers (CHWs) in the COVID-19 response is widely recognized, evidence shows they often lacked clear guidance on their responsibilities despite their close ties to communities [14]. Indeed a study on CHWs’ experiences during COVID-19 in six countries found that support for their pandemic response varied both across and within countries [18]. Significant gaps were identified, including disrupted medical supply chains and high workloads, which left CHWs vulnerable to infection and stress [18].

Despite policy guidance promoting the involvement of CHWs in the COVID-19 response, there remains a lack of comprehensive evidence synthesis regarding their specific roles, challenges, and strategies for enhancing their performance [19]. Existing reviews have not addressed all three dimensions simultaneously [18, 20–23]. This gap hinders the development of resilient CHSs for future infectious outbreaks [19].

Our study aimed to synthesize literature on the roles and challenges faced by CHWs in combating COVID-19, and to propose strategies for enhancing their performance. We adopted a scoping review methodology, which is particularly useful for identifying the types of evidence available in emerging fields—such as that surrounding the COVID-19 pandemic [24].

## Methods

### The search strategy

We systematically searched databases for literature on the roles, responsibilities, and challenges faced by community health workers (CHWs) during the COVID-19 pandemic. We also looked for strategies and support structures designed to address these challenges. The databases included PubMed, HINARI, Cochrane Library (Reviews and Trials), Science Direct, and Google Scholar.

Between January and July 2023, we conducted searches using terms related to “community health workers (CHWs)” and “COVID-19” or “COVID-19 Vaccine.” For CHWs, we included terminology outlined in a systematic review of CHW definitions. These terms included: Accredited Social Health Activist, Lady Health Worker, Community Health Advisor, Patient Navigator, Lay Health Worker, Community-Based Health Provider, Peer Educator, Community Health Representative, Care Facilitator, Community Health Agent, Community-Based Reproductive Health Agent, Auxiliary Nurse Midwife, Village Health Worker, Health Extension Worker, Lay Health Promoter, Care Guide, Peer Health Advisor, Community Health Development Agent, Community Health Promoter, Lay Health Educator, Community-Based Health Worker, Community Health Coach, Village Health Volunteer, Community Midwife, Community Health Assistant, Community-Based Educator, and Health Surveillance Assistant [25].

### Inclusion and exclusion criteria

We included only English-language publications. To ensure the inclusion of relevant, high-quality papers, our criteria focused on peer-reviewed publications, as well as reports and guidelines from the WHO and United Nations organizations related to the study topic.

Eligible studies were conducted between December 2019 and July 2023, which represents the period of heightened COVID-19 activity. Given that this is a scoping review, we included papers with various study designs. These included qualitative, quantitative, and mixed-methods studies, as well as reviews, CHW program evaluations, reports, and commentaries.

We excluded studies on COVID-19 that did not discuss CHW roles, challenges, or strategies for improving CHW performance. We also excluded studies conducted in high-income countries.

### Study selection and quality assessment

**Fig. 1:**
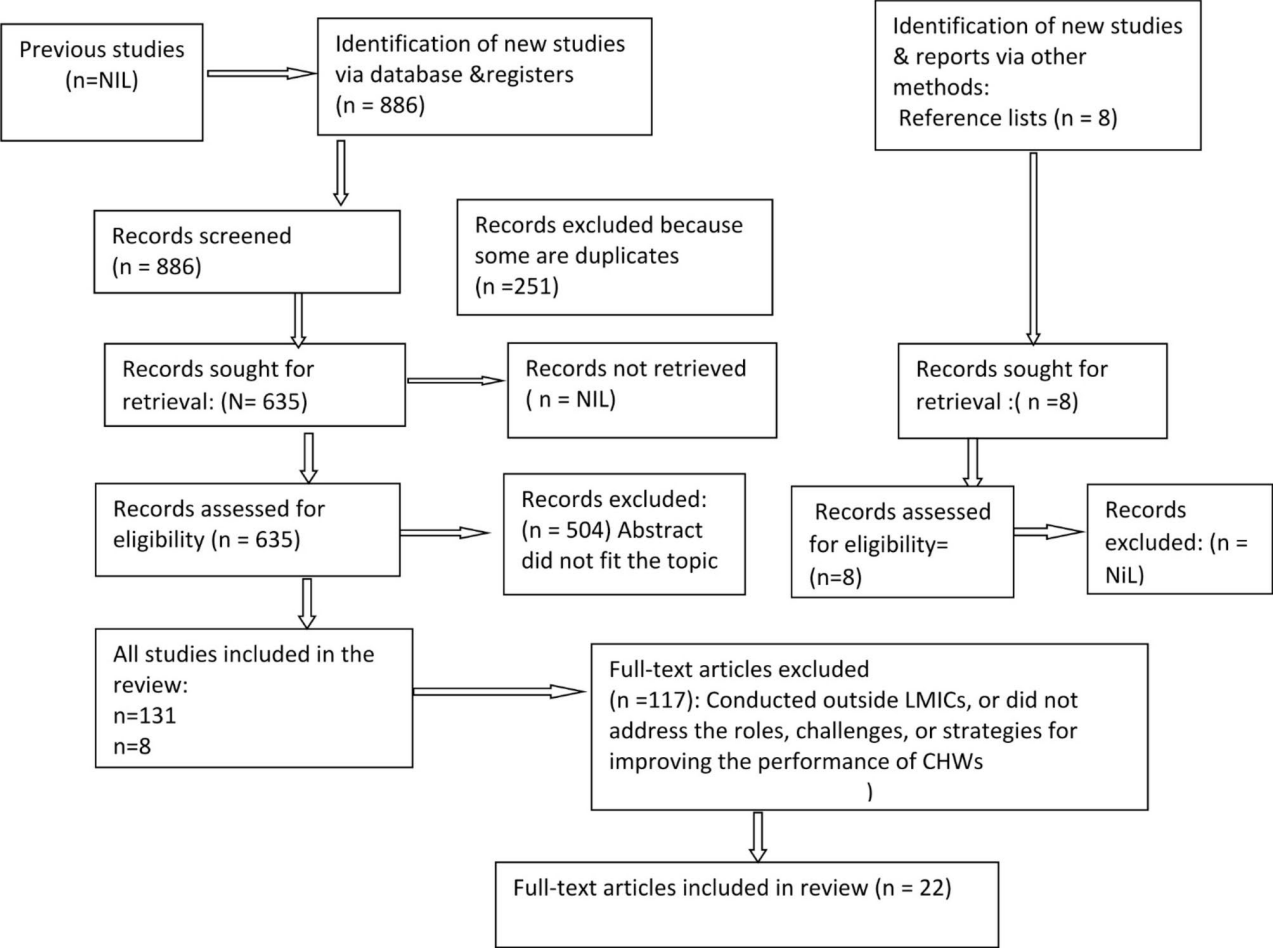
PRISMA flow diagram of scoping review

We followed the Preferred Reporting Items for Systematic Reviews and Meta-Analyses (PRISMA) guidelines for selecting studies (Fig. 1) [26]. In line with these guidelines, we first excluded 251 duplicates from the initial 894 search results (886 identified through databases/registers and 8 through reference lists). We then reviewed the titles of the remaining studies and excluded 504 that focused on the wrong topic, region, or both.

Next, we retrieved and assessed the abstracts of 139 papers. We excluded 117 that did not address the roles, challenges, or strategies for improving CHW performance in preventing and managing COVID-19.

At this stage, we also applied the Critical Appraisal Skills Program (CASP) quality assessment, particularly for qualitative studies [27]. This process resulted in a final selection of 22 papers for analysis (Fig. 1 and Supplementary File 1).

### Data analysis and synthesis

We conducted a thematic analysis using NVivo 12 Pro Software (QSR International, Melbourne, Australia) [28]. The final full-text articles were imported into NVivo for coding. A coding framework was developed to guide the process, based on the study’s main objective. This framework included broad and sub-themes, focusing on three key areas: roles, challenges, and strategies related to CHW performance in the COVID-19 response in low- and middle-income countries (Table 1).

**Table 1:** Key thematic categories from the literature

Main Themes	Sub-themes
Roles for community health workers	Health promotions and education
	Assisting with surveillance
	Maintaining essential health services
	Support planning and coordination of vaccination
The challenges faced by CHWs during the COVID-19 response	Stigma and discrimination
	Limited incentives
	The lack of training for CHWs during the COVID-19 response
	Inadequate infection prevention and control preparedness
	Limited supportive policies
Strategies for enhancing the performance of CHWs during COVID -19	Harnessing digital technology (mHealth)
	Training for CHWs in pandemic response
	Health information management: collection and dissemination
	Wellness and safeguarding CHWs

We first reviewed the abstracts of the selected articles to inform the coding framework. The codes were then discussed by the review team, and a final set was agreed upon for use in the analysis. This finalized framework was imported into NVivo for detailed coding.

Two research team members, Adam Silumbwe (AS) and Malizgani Paul Chavula (MPC), carried out the coding. They regularly updated the full team on their progress. An iterative grouping of the codes was then conducted to identify patterns and generate sub-themes within the three broad themes.

A draft of the main themes and sub-themes was shared with all co-authors. This allowed for discussion, clarification, and identification of linkages across themes. After finalizing the process, we retained the three predetermined themes and identified 12 sub-themes (Table 1). These formed the basis for drafting the findings.

The draft findings were reviewed and agreed upon by all co-authors, leading to the results presented in this article.

## Results

In this section, we present the findings from published studies on the involvement of CHWs during the COVID-19 pandemic (Table 2). We begin by outlining the roles CHWs played in response to the pandemic, followed by a discussion of the challenges they encountered while fulfilling these roles. The final part of this section details strategies for enhancing CHW performance during the pandemic.

**Table 2:** Study characteristics: A scoping review of the roles, challenges, and strategies for enhancing the performance of community health workers in the response against COVID-19 in low-and middle-income countries

No	1^st^ Author/ Year/ Journal	Study type/design / Site/s	Study title/aim	Study participants/ Primary source of information	Key issues/findings
1	Lotta G, et al., (2021) *Journal of Comparative Policy Analysis: Research and Practice 23.1 (2021): 63–73*.	Document Review Brazil	How COVID-19 has affected frontline workers in Brazil: a comparative analysis of nurses and community health workers. To analyse how the Brazilian government regulated the reorganization of Primary Health Care (PHC) and how frontline workers responded to these initiatives, comparing the roles played by nurses and community health workers.	Documents	• Given the multilevel health system, it was expected that the high level of ambiguity would stimulate innovations. • However, data showed that the ambiguity created different situations for each profession. • While nurses were able to adapt their work and act with more autonomy, CHWs lost their role in the policy.
2	Ballard et al., (2020) *BMJ global health*,* 5(6)*,* e002550.*	Document Review Low and middle income countries	Prioritising the role of community health workers in the COVID-19 response. Examining prioritising the role of community health workers in the COVID-19 response.	Documents	• Community health workers (CHWs) are poised to play a pivotal role in fighting the pandemic, especially in low-income countries with vulnerable health systems. • The COVID-19 response must build on existing platforms, infrastructure, and relationships; the focus should be on supporting the Ministries of Health and regional authorities as they lead coordinated responses. • Achieving these goals will require targeted actions at different stages of the pandemic.
3	Ajisegiri, et al., (2020) *Global Biosecurity. 2020;2(1).*	Systematic review Nigeria	COVID-19 outbreak situation in Nigeria and the need for effective engagement of community health workers for epidemic response. Examining COVID-19 outbreak situation in nigeria and the need for effective engagement of CHWs for epidemic response.	Published articles from the website of the Nigeria Centre for Disease Control	• Recommended that the government needs to promptly bring community health workers on board, deploy rapid epidemic intelligence and scale up the use of mobile Apps for contact tracing. • These changes will result in an effective and coordinated response to the outbreak, sustain routine health services especially at the community level.
4	Bezbaruah et al., (2021) *Journal of Public Health. 2021;10(3):41.*	Rapid review of documents South-East Asia	Roles of community health workers in advancing health security and resilient health systems: emerging lessons from the COVID-19 response in the South-East Asia Region. WHO South-East Asia. To explore the roles of community health workers in advancing health security and resilient health systems: emerging lessons from the COVID-19 response in the South-East Asia Region.	Review journal articles, policy documents, national guidelines, reports, and online publications	• The regular role of a CHW in health education and promotion focused on awareness-raising and the promotion of “new normal” behaviours. CHWs also played critical roles in assisting in surveillance and contact tracing, and in ensuring that people followed isolation and quarantine guidelines. • Development and implementation of long-term plans across the region to strengthen and support CHWs and recognize CHWs as an integral component of resilient health systems. • Planning for CHWs as part of the primary health care system will enable local authorities to ensure that an adequate level of resources (including capacity-building, incentives, necessary equipment, and consumables) is allocated to CHWs.
5	Mistry et al. (2021) *Frontiers in Public Health. 2021:800.*	Document Review Low and middle income countries	Community health workers can provide psychosocial support to the people during COVID-19 and beyond in low-and middle-income countries. To explore community health workers’ role in providing psychosocial support to the people during COVID-19 and beyond in low-and middle-income countries.	Documents	• The CHWs can be effectively engaged to provide psychosocial support at the community level. Engaging them can also be cost saving as they are already in place and may cost less compared to other health professionals. However, they need training and supervision and their safety and security need to be protected during this COVID-19. • While many LMICs have mental health policies, their enactment is limited due to the fragility of health systems and limited health care resources. • CHWs can contribute in this regard and help to address the psychosocial vulnerabilities of affected population in LMICs during COVID-19 and beyond.
6	Roy et al., (2020) *Global Health: Science and Practice. 2022;10(4).*	Mixed methods Bangladesh	Examining Roles, Support, and Experiences of Community Health Workers During the COVID-19 Pandemic in Bangladesh: A Mixed Methods Study. Examining roles, support, and experiences of community health workers during the COVID-19 pandemic in Bangladesh.	Policy makers, program managers, CHW supervisors, and CHWs	• During the first wave of the coronavirus disease (COVID-19) pandemic in Bangladesh, across all health areas, community health workers (CHWs) described a slight decrease in the routine services they were able to provide due to restrictions in movement posed by lockdowns and other challenges. • The government and various nongovernmental organizations provided supportive mechanisms to CHWs through training, supplies, and supportive supervision; however, this support were not always uniformly distributed across cadres, leading to some discontent among CHWs. • CHWs were crucial actors in the government’s COVID-19 response, as they took on new pandemic-related responsibilities in their communities to prevent the spread of the disease while continuing their routine work.
7	Fernandez et al., (2020) *Archive of Family Medicine and General Practice. 2020;5(1):115 − 22*	Quantitative Brazil	How community health workers are facing COVID-19 pandemic in Brazil: personal feelings, access to resources and working process. To examine how community health workers are facing COVID-19 pandemic in Brazil.	Online Survey	• CHWs feel scared and unprepared in the face of the COVID-19 pandemic. The fear of COVID-19 is related to being prepared and to receiving support from federal government. The feeling of preparedness is associated with the lack of material working conditions, such as PPEs, guidance from managers and support from superiors and federal government.
8	Nepomnyashchiy et al., (2020) *The Lancet. 2020;396(10245):150-2.*	Review of documents Africa	COVID-19: Africa needs unprecedented attention to strengthen community health systems. To discuss the importance of unprecedented attention to strengthen community health systems during the COVID-19 pandemic.	Review of documents	• CHWs matter because they are trusted members of the community who are often the most accessible point of care. • Ongoing efforts to leverage CHWs for the COVID-19 response must not be one-off in the face of an emergency. CHWs must be equipped, trained, and supported in the long term as a crucial human resource for health.
9	Maciel FBM., (2020) *Ciência & Saúde Coletiva. 2020;25:4185-95.*	Qualitative study	Community health workers: reflections on the health work process in Covid-19 pandemic times.	Literature review	• CHW work, especially cultural competence, and community orientation, aiming to discuss the changes introduced in this work regarding the following aspects: (1) health teams support, (2) use of telehealth, and (3) health education.
10	Chitungo et al., (2021) *Human behavior and emerging technologies. 2021;3(5):843 − 53.*	A rapid review Sub-Saharan Africa	Utility of telemedicine in sub-Saharan Africa during the COVID‐19 pandemic. A rapid review. The article aimed to propose the development of policy frameworks that fosters telemedicine use by all stakeholders.	A rapid review	• Challenges to the implementation of telemedicine on the continent were lack of supporting telemedicine framework and policies, digital barriers, and patient and healthcare personnel biases. • To enhance the use of telemedicine, policies should be developed to support the use of telemedicine by all stakeholders, including medical insurance organizations, the introduction of telemedicine training of medical workers, educational awareness programs for the public, and improvement of digital platforms access and affordability.
11	Feroz et al.,. (2021) *Archives of Public Health. 2021;79(1):1–4.*	Review of documents	Equipping community health workers with digital tools for pandemic response in LMICs. The article aimed to discuss ways of supporting CHWs with digital technology to ensure an appropriate pandemic response.	Review of documents	• CHWs are playing a huge role in providing essential health care services and Covid-19 related healthcare to the communities. • CHWs are overburdened as they are expected to accomplish more although they are not getting the required support to perform their duties well, such as training, remuneration, protective gear, etc.
12	Bhaumik et al., (2020) *BMJ Global Health. 2020;5(6):e002769.*	Systematic review Low-and middle-income countries	Community health workers for pandemic response: a rapid evidence synthesis The review aimed to conduct a rapid evidence synthesis on community health workers (CHWs) for COVID-19 prevention and control.	Articles	• CHW roles and tasks change substantially during pandemics. Clear guidance, training for changed roles and definition of what constitutes essential activities (i.e., those that must be sustained) is required. • Most common additional activities during pandemics were community awareness, engagement, and sensitisation (including for countering stigma) and contact tracing. • CHWs were reported to be involved in all aspects of contact tracing - this was reported to affect routine service delivery. CHWs have often been stigmatised or been socially ostracised during pandemics.
13	Sudhipongpracha et al., (2020) *Journal of Comparative Policy Analysis: Research and Practice. 2021;23(2):234 − 49*.	Qualitative study Kenya and Thailand	Community health workers as street-level quasi-bureaucrats in the COVID-19 Pandemic: The cases of Kenya and Thailand. This article used cross-country comparative analysis to explore how community health workers (CHWs) deal with the COVID-19 pandemic.	Semi structured interviews	• Findings show that how a public health system is organized (decentralization versus centralization) affects CHWs’ initial responses to the outbreak. • While CHWs in Thailand’s centralized system conform to the “state agent” tradition by referring to the hierarchical chain of command, those in Kenya’s decentralized system follow the “citizen agent” tradition by prioritizing community safety.
14	Jalali, F., Fischer, H., & Nichols, C. (2022). *Political Geography*, *99*.	Qualitative study India	Corona warriors”? Experiences of India’s community health workers (ASHAs) in India’s COVID-19 response. This paper aimed to explore the ways that nationalist COVID-19 war rhetoric promulgated from Delhi worked as a technology of health governance to propel ASHAs into certain forms of action.	In-depth telephone interviews with CHWs	• CHWs (ASHAs) were both proud ‘warriors’ and compelled to work due to the risk of letting down their community. • While many CHWs however felt deep fear and that they were ill-prepared. • CHWs reported that they made sacrifices both to their own personal health as well as their families.
15	Niyigena, A., Girukubonye, I., Barnhart, D. A., Cubaka, V. K., Niyigena, P. C., Nshunguyabahizi, M.,… & Bitalabeho, F. A. (2022). BMJ open. 2022;12(4):e055119.	Mixed-method study Rwanda	Rwanda’s community health workers at the front line: a mixed-method study on perceived needs and challenges for community-based healthcare delivery during COVID-19 pandemic. The paper aimed to understand challenges faced by Rwanda’s CHWs during a nationwide COVID-19 lockdown.	CHWs	• Supervision during the lockdown was low. • CHWs additionally described increases in workload, lack of personal protective equipment and COVID-specific training, fear of COVID-19, and difficult working conditions.
16	Sripad, P., Gottert, A., Abuya, T., Casseus, A., Hossain, S., Agarwal, S., & Warren, C. E. (2022). *PLOS global public health 2*,* no. 10 (2022): e0000595*.	This mixed methods Bangladesh, Haiti and Kenya	Confirming—and testing—bonds of trust: A mixed methods study exploring community health workers’ experiences during the COVID-19 pandemic in Bangladesh, Haiti and Kenya. The study explored trust through the evolving COVID-19 crisis in Bangladesh, Haiti, and Kenya.	Interviews with CHWs	• CHWs reported high levels of community trust (8/10 in Bangladesh and Kenya; 6/10 in Haiti). • Over 60% reported client relief in seeing their CHWs. • CHWs reporting more positive and fewer negative experiences is consistently associated with continuing routine work, doing COVID-19-related work, and greater community trust. Qualitative interviews showed that CHW-community and CHW-health system actor trust is strengthened when CHWs are well-resourced. • CHW-community trust is strained by public frustration at the pandemic, associated restrictions, and socio-political stressors.
17	Dhaliwal, B. K., Singh, S., Sullivan, L., Banerjee, P., Seth, R., Sengupta, P.,… & Shet, A. (2021). *Journal of global health*, *11*.	Rapid qualitative evaluation India	Love, labor and loss on the frontlines: India’s community health workers straddle life and the COVID-19 pandemic The study aimed to understand how the pandemic impacted the professional and personal experiences of CHWs in India during the pandemic.	Interviews with CHWs	• CHWs faced increased workloads, decreased compensation, and stated that their work had shifted to focus on COVID-related work, as opposed to routine care. • CHWs also shared that their needs included improved mental health services, financial payment that was not tied to incentives, and consistent access to PPE. • CHW experiences through the context of the COVID-19 pandemic have not been well-explored.
18	Monreal, T. J., Falcão de Oliveira, E., Araujo Ajalla, M. E., Adania Zanoni, D., & Du Bocage Santos-Pinto, C. (2022). *Sociological Spectrum*, *42*(3), 217–230.	A descriptive cross-sectional study- quantitative Brazil	Community health workers and COVID-19 in a Brazilian state capital. The aim of this study was to determine the COVID-19-related health status of CHWs, their basic knowledge of the disease and the role they played in the pandemic response.	Questionnaires with CHWs	• Around 40% of the sample reported at least one risk factor for COVID-19, 44% had experienced at least one COVID-19 symptom, and 76% had experienced symptoms of mental suffering during the first year of the pandemic. Mental suffering was associated with the onset of flu-like symptoms after the start of the pandemic and changes in work processes. Knowledge gaps were observed, mainly related to forms of transmission and disease prevention. In view of the uncertainty about how long this health emergency will last and the vital role CHWs play in the Brazilian Health System, health managers and society need to pay greater attention to these professionals to improve the effectiveness of the country’s COVID-19 response.
19	Gibson, E., Zameer, M., Alban, R., & Kouwanou, L. M. (2023). *Global Health: Science and Practice*,* 11(1).*	A rapid review Global landscape	Community health workers as vaccinators: a rapid review of the global landscape, 2000–2021. This rapid review aimed to identify countries where CHWs administered vaccines and synthesize health systems factors that may contribute to or detract from the feasibility of CHWs administering vaccines.	Peer-reviewed literature	• Community health worker (CHW) cadres administered vaccines in 20 of the 75 countries with documented CHW programs, improving access to immunization services for under-reached communities. • The review identified several countries where CHWs with brief clinical training and experience were taught to vaccinate, suggesting the feasibility of task-shifting administering vaccines to CHWs with limited experience.
20	Olateju e al., (2022). *PloS one. 2022;17(3):e0265092.*	Qualitative study Nigeria	Community health workers experiences and perceptions of working during the COVID-19 pandemic in Lagos, Nigeria—A qualitative study. To explore community health workers’ experiences and perceptions of working during the COVID-19 pandemic in Lagos, Nigeria	Interviews with CHWs	• Trust and COVID-19 knowledge were found to aid CHWs in their work. However, challenges included exhaustion due to an increased workload, public misconceptions about COVID-19, stigmatisation of COVID-19 patients, delayed access to care and lack of transportation. • *Influences on willingness to work in COVID-19* included CHWs ’ perceptions of COVID-19, attitudes towards responsibility for COVID-19 risk at work, commitment and faith. • Financial incentives, provision of adequate personal protective equipment, transportation, and increasing staff numbers were seen as potential strategies to address many of the challenges faced.
21	Salve, S., Raven, J., Das, P., Srinivasan, S., Khaled, A., Hayee, M.,… & Gooding, K. (2023). *PLOS Global Public Health*,* 3(1)*,* e0001447.*	Synthesis of evidence India, Bangladesh, Pakistan, Sierra Leone, Kenya and Ethiopia	Community health workers and Covid-19: Cross-country evidence on their roles, experiences, challenges and adaptive strategies. The paper aimed contribute to learning about CHWs’ experiences during COVID-19, based on evidence from India, Bangladesh, Pakistan, Sierra Leone, Kenya and Ethiopia.	Synthesises evidence from a set of research projects	• CHWs made important contributions to the COVID-19 response, including in surveillance, community education, and support for people with COVID-19. • There was some support for CHWs’ work, including training, personal protective equipment, and financial incentives. • However, support varied between countries, cadres and individual CHWs, and there were significant gaps, leaving CHWs vulnerable to infection and stress. • CHWs also faced a range of other challenges, including health system issues such as disrupted medical supply chains, insufficient staff and high workloads, a particular difficulty for female CHWs who were balancing domestic responsibilities. • CHWs demonstrated commitment in adapting their work, for example ensuring patients had adequate drugs in advance of lockdowns and using their own money and time to address increased transport costs and higher workloads.
22	World Health Organization. (2021). *World Health Organization Website*	Report Global landscape	The role of community health workers in COVID-19 vaccination. The guide intended to support national governments in developing their national deployment and vaccination plans (NDVP) for COVID-19 vaccines by outlining the roles, needs and opportunities for (CHWs).	Reports and articles	• This guide is intended to support national governments in developing their national deployment and vaccination plans (NDVP) for COVID-19 vaccines by outlining the roles, needs and opportunities for community health workers (CHWs). • Identifying CHW contributing roles at each stage of COVID-19 vaccines rollout. • Counting and vaccinating CHWs within initial vaccine allocation as part of the essential health • Workforce to optimally support the COVID-19 response and continuity of essential health services. • Recognizing and remunerating CHWs commensurate to tasks undertaken and training. • CHWs who are linked to health systems through regular compensation, dedicated supervision and accreditation are best placed to support an effective pandemic response and to prevent the next one. • Considering community-based health worker representation on national coordinating committees.

Twelve [12] articles were reviews, five [5] qualitative studies, and three [3] quantitative studies while two [2] studies used mixed method designs. The articles that used primary data sources were conducted in Africa, Asia and South America (Table 2).

### Roles for community health workers

#### Health promotion and education

CHWs played a key role in COVID-19 related health promotion and education activities [29]. In India, Bangladesh, Kenya and Ethiopia, they helped promote the acceptability of COVID-19 prevention measures by first adopting these measures themselves, such as wearing masks and practicing physical distancing [30]. They also played a critical role in delivering culturally sensitive information to counter practices, social norms and misinformation that could facilitate the spread of the virus [30]. This included dispelling myths, such as the belief that COVID-19 could not be transmitted in hot and humid climates, that mosquito bites could spread the virus, or that the disease only affected certain groups of people [30].

By providing health education on the nature and prevention of COVID- 19, CHWs helped reduce social stigma or superstitions associated with the disease. They also contributed to preventing discrimination against patients and their families [18]. Additionally, CHWs acted as role models, and behaviour change agents by accepting vaccines and getting vaccinated ahead of other community members when vaccines became available [18].

CHWs used various strategies to improve access to information on COVID-19 prevention [31]. These included home visits and public information-sharing initiatives, such as using megaphones, as observed in Kenya, India, Thailand, Ethiopia, and Indonesia [29, 32].

In some countries, health promotion was integrated into national CHW guidelines, as seen in India and Thailand [29]. In Bangladesh, CHWs acted as a bridge between refugee communities and health facilities, helping to address fears and dispel rumours [33].

#### Assisting with surveillance

As permanent residents within their communities, CHWs played a crucial role in supporting disease surveillance activities, such as contact tracing and enforcing quarantine directives [29]. They were often described as “natural researchers”, for example, countries like Kenya, Liberia, India, and Rwanda leveraged CHWs for COVID-19 case detection [18]. In Bangladesh, India, Nepal, and Thailand, CHWs conducted symptomatic screenings to detect COVID-19 infections. In India, as internal migrants returned home after the lockdown, CHWs screened 30 to 50 households per day for symptoms [18, 29].

CHWs played a key role by supporting the reintegration of recovered patients into their communities [18]. They successfully reduced the stigma associated with recovered individuals by promoting voluntary quarantine in dedicated facilities. CHWs utilized their local knowledge to implement safety measures effectively during surveillance activities. In India, Bangladesh, and Ethiopia, they facilitated the willingness of symptomatic family members to agree to admission to treatment centers [18]. Furthermore, through community collaboration, the CHWs achieved significant progress by educating individuals about quarantine protocols and effectively identified those exposed to the virus [18]. By so doing, they significantly reduced stigma against those who had recovered as well as provided more information on causes, prevention and effects of COVID-19 spread from recovered identities [18].

#### Maintaining essential primary health care services

CHWs played a critical role in distributing essential household products and medical supplies to individuals in self-isolation. They also coordinated transport and lodging for vaccinators and identified outreach locations to reach vulnerable populations. In countries like Bangladesh, Haiti, and Kenya, CHWs leveraged trusted networks to support both COVID-19-related and unrelated health services, including referrals for maternal health services [30, 33]. In Malawi, CHWs engaged in social mobilization to promote HPV vaccines when schools closed during the pandemic [18, 23].

While CHWs were essential in maintaining established health services, it is important to recognize the emergence of new healthcare needs during the pandemic [29]. One of the most notable was the increased demand for mental health services worldwide due to the pandemic’s impact [34]. In response, CHWs provided crucial psychosocial support in countries such as India, Uganda, Nepal, and Pakistan [29, 34]. The pandemic led to a sharp rise in stress, anxiety, fear, depression, and anger, creating an expanded role for CHWs to address mental health concerns within their communities [34, 35].

#### Support planning and coordination of vaccination

The involvement of CHW in vaccination planning teams was crucial in identifying target or priority populations by mapping out vaccination locations [36]. In Pakistan, for instance, CHWs registered households to ensure accurate forecasting, mobilized target populations, and accompanied them to immunization sites [23]. They also promoted the COVID-19 vaccine by delivering relevant, context-specific information during the preparatory and planning stages [23]. Furthermore, CHWs enhanced vaccine acceptance by engaging community influencers, acting as a link between the community and vaccination centers, and supporting the scheduling process. They also organized the flow of vaccine recipients, both in person and via teleconsultation [30].

### The challenges faced by CHWs during the COVID-19 response

#### Stigma and discrimination

The interaction of CHWs with individuals infected with COVID-19 exposed them to both stigma and the virus itself. In India, for instance, a group of people assaulted CHWs who were collecting data on individuals with COVID-19-like symptoms [29]. Many CHWs felt scared and unprepared due to a lack of protective equipment. In Nigeria, social stigmatization of COVID-19 patients led many individuals to conceal their infection, making it difficult for CHWs to identify cases within the community [37].

#### Limited incentives

India, Bangladesh, Pakistan, Sierra Leone, Kenya, and Ethiopia, faced challenges with the regular payment of adequate remuneration and incentives for CHWs during the pandemic [18, 38]. Without a more harmonized approach to CHW compensation, their motivation and performance in COVID-19 prevention efforts became inconsistent [38]. In India, Bangladesh, and Pakistan, additional financial incentive schemes were introduced to compensate CHWs for the increased workload and risks related to COVID-19 [18]. However, despite these additional incentives, gaps remained, disrupting routine service delivery [18].

Further, in some cases (in India), the CHWs were unaware of these additional incentives, while in some cases, they were not paid at all [18]. There were also disparities between CHW cadres, leading to demotivation, with some CHWs going on strike due to the lack of incentives [18]. In Ethiopia, CHWs often spent their own money to provide services during the pandemic without reimbursement [18]. In Nigeria, the lack of transportation for CHWs in rural areas during lockdowns hampered their ability to perform services [30]. Overall, precarious remuneration impacted CHWs’ ability to deliver essential services, particularly in India [18, 37, 39, 40].

#### The lack of training for CHWs during the COVID-19 response

The type and level of COVID-19 training provided to CHWs varied and was often inadequate, irregular, and inappropriate [38]. In Brazil, while CHWs played essential roles in fighting the pandemic, early response and capacity building efforts focused primarily on the frontline health workers, neglecting the CHWs. As a result, CHWs were not initially oriented on their roles in pandemic control, and little was done to protect them from COVID-19 while performing these duties in the community [15]. Given that CHWs significantly outnumber health workers, this oversight was seen as a missed opportunity in the early stages of the pandemic response [41].

#### Inadequate infection prevention and control preparedness

At the onset of the pandemic, CHWs in Kenya and Thailand faced a significant dilemma– the gap between the availability of resources and what needed to be done to prevent the outbreak [20]. Despite government-issued non-pharmaceutical intervention guidelines, CHWs in both countries reported a lack of necessary resources and equipment. When the national governments in Kenya and Thailand made it compulsory to wear masks in public, shortages of face masks and N95 respirator masks quickly followed. Funding for personal protective equipment (PPE) and related supplies, such as face masks, soaps, and hand sanitizers, posed a significant challenge for CHWs in Kenya [20].

In Rwanda, the lack of PPE coupled with inadequate COVID-19-specific training and increased workloads affected CHW’s ability to deliver services during the pandemic [42]. Similarly, the lack of appropriate technology to conduct health education posed a challenge [21]. Given the highly infectious nature of the COVID − 19, the lack of tools and systems that allowed CHWs to provide health education with minimal contact negatively affected their ability to deliver health messages [43]. In instances where such tools and systems existed, limited knowledge and capacity hindered the CHWs ability to use them effectively [32, 43].

### Limited supportive policies

At the beginning of the pandemic, there was a clear lack of sufficient guidelines to support CHW participation in COVID- 19 prevention services were evident [20]. The novel and rapidly evolving nature of COVID − 19 made it even more difficult for policymakers to craft timely, appropriate and responsive policies [20].

In Kenya and Thailand, the CHWs highlighted the ambiguity and uncertainty of the policy environment [20]. Additionally, in countries like Thailand, India, Bangladesh, Pakistan, Sierra Leone, Kenya and Ethiopia, there were no clear policy guidelines with regard to CHW roles in preventing COVID − 19, making referrals, supporting vaccination efforts, or accessibility to prevention materials [20].

The absence of such guidelines hindered CHWs ability to effectively deliver primary health care. This included challenges in health promotion, surveillance, contact tracing, quarantine enforcement, and maintenance of essential health services [20].

Interestingly, despite the lack of formal guidelines at the start of the pandemic, CHWs in Kenya reported relying on protocols used during the Ebola epidemic. The urgency of the situation compelled them to draw on this experience to prevent the outbreaks in their communities [20].

### Mental health challenges

Some CHWs in India, Bangladesh, Pakistan, Sierra Leone, Kenya and Ethiopia experienced mental distress and anxiety while delivering services during the COVID-19 pandemic [18]. These mental challenges were stemmed from discrimination and stigma from both the community and family members. Some CHWs were discriminated against because people believed they had COVID − 19 and could transmit it to others. Distress and anxiety was also caused by heavy workloads and an increase in deaths from COVID-19 related diseases [18].

### Strategies for enhancing the performance of CHWs during COVID-19

#### Training for CHWs in pandemic response

Several approaches were recommended and, in some instances, adopted to help CHWs to adapt and continue work during the COVID-19 pandemic. In Brazil and India, organized health education and orientation were both implemented and recommended as part of the initial pandemic response [22]. It was noted that such education, coupled with culturally accessible communication mechanisms was crucial in effectively fighting COVID-19 and future pandemics. Given that most emerging infectious diseases are zoonotic in origin, training CHWs to communicate ‘One health’ information to at-risk communities prior to outbreaks may enhance future pandemic preparedness [39, 44].

#### Health information management: collection and dissemination

While health information dissemination is seen as a major gap that CHWs can address, as demonstrated in Bangladesh, their close contact with community members also offers a valuable opportunity. They are well-positioned to gather context-specific misinformation and misconceptions from the public, which can then be directly targeted through health messaging [41].

The COVID-19 pandemic experience underscored the importance of effective information management. CHWs played a crucial role in correcting of myths and misconceptions, while also disseminating accurate and reliable information to communities [44].

#### Harnessing digital technology (mHealth)

Harnessing digital technology (mHealth) was one of the strategies used to support CHWs during the COVID-19 pandemic response to enhance their performance [21]. This included the use of short message service (SMS) and voice messages for health education, digital megaphones to encourage behavior change, digital contact tracing and case recording, as well as mHealth platforms for CHW education, training and supervision [21, 43].

For example, CHWs in Uganda and Ghana established collaborative groups via mobile-messaging apps such as WhatsApp [21, 43]. CHWs could access the WHO COVID-19 online training resource information through their mobile phones [21, 43].

However, more work is needed to improve the overall feasibility and acceptability of digital tools for CHWs. Many CHWs are inadequately trained in using these tools and may face challenges such as weak technical support and poor internet connectivity [32]. Despite these limitations, countries like Uganda and Ethiopia have successfully implemented digital platforms, providing valuable lessons and best practices for future pandemic responses [21, 43].

However, more work is needed to improve the overall feasibility and acceptability of digital tools for CHWs. Many CHWs are inadequately trained in using these tools and may face challenges such as weak technical support and poor internet connectivity [32]. Despite these limitations, countries such as Uganda and Ethiopia successfully implemented digital platforms, providing valuable lessons and best practices for future pandemic response [21, 43].

#### Wellness and safeguarding CHWs

While CHWs are essential in serving and protecting vulnerable populations during crises, their own health and wellbeing—including mental health—were also at risk. This was largely due to insufficient motivation, inadequate remuneration, and limited protection against the pandemic [20]. It is therefore important to implement wellness programs for CHWs. These should include access to adequate and quality protective equipment, as well as peer support programs for mental health [20]. To ensure these strategies are effective, supportive policies and regulations must be developed that recognize the role of CHWs in responding to the COVID-19 pandemic.

Such policies could include integrating CHWs into a reserve health corps for public health emergencies. They could also involve formal agreements to promote CHW engagement in response efforts. Like other healthcare providers, CHWs should have been prioritized for early access to COVID-19 vaccinations. They should also have received regular training to stay aligned with the evolving nature of pandemics.

Additionally, providing adequate institutional support can strengthen the trust between CHWs and the communities they serve. This would also promote more resilient community health systems (CHSs) during public health crises [33].

## Discussion

We observed that CHWs played many roles during the COVID-19 response. These included serving as mobilizers, role models, promoters for behavioural change, providers of essential services, and surveillance personnel. CHWs provided valuable support to health systems during the pandemic, as they had unique capabilities which formal health workers lacked. For example, as trusted and valued members, CHWs could easily navigate through the community, help address myths and misconceptions regarding the pandemic and successfully manage referrals.

Historical experience indicates that community actors have played a key role in delivering health messages that promote vaccine acceptance across the globe during pandemics [45, 46]. Indeed, by advocating for and countering the widespread scepticism with accurate information, CHWs are crucial in promoting community acceptance of vaccines [4, 47].

We note that through performing these roles, CHWs could contribute towards equitable universal primary health care attainment during pandemics [45, 46]. This is especially important given their unique capabilities to promote health and deliver services and information to remote areas and underserved populations [46]. Additionally, being situated within their communities, they have earned the trust of those they serve [4, 47].

Trust is an important capability that CHWs can leverage on to address disinformation and misinformation. These often thrive on mistrust of formal authorities such as governments and health workers during pandemics [4, 47]. Further, the endorsement and support of CHWs by community leaders [48, 49], give their services legitimacy. This is vital in enhancing the acceptance of primary health care services offered during pandemics, either as preventative or curative [48, 49]. Support from community leaders also provides an additional communication channel through leaders themselves or other community-level communication systems, thereby amplifying CHWs’ messages [50].

A combination of CHW trust and community leadership support during the pandemics can simultaneously contribute to building resilient CHSs [51]. Nurturing such trust could also trigger a sense of community and shared responsibility, which, according to WHO, was critical in the fight against COVID-19 [52]. Against the reality of present and future “infodemics”, we thus suggest that the effective use of CHWs will be critical. They can enhance the benefits of a multifaceted approach to communicating behavioural change messages for future pandemics and maintaining delivery and accessibility to primary health care services [20, 41].

We have also documented that performing these roles was met with challenges [29]. These included, ambiguity and stigma from the community members, the lack of adequate training, inadequate infection prevention and control preparedness, lack of supplies and commodities, limited supportive policies and inadequate remuneration and incentives [29]. These challenges affected their performance by exposing them to infection risks, limiting their coverage and capacity to deliver information and services [29].

While some challenges, such as COVID-19-related stigma and discrimination, were new, others like inadequate incentives are historical and ongoing issues for CHWs [29]. These challenges are largely due to inadequate prioritisation and integration of CHW incentives into the health systems [53]. The stress that COVID-19 placed on health systems worsened this situation, as CHWs had to work more to fill human resource gaps. Competing health needs led some health systems to neglect CHW financial incentives [18].

Addressing these challenges will require integrating standardised incentives for CHWs within the national budget in line with WHO recommendations [54]. According to WHO, governments should provide financial packages that reflect CHWs’ job demands, complexity, hours worked, training, and roles [53]. It is important that CHWs are not viewed as panacea for weak health systems [55].

Overall, there has been an increased call for health systems in LMICs to invest in CHWs. This is vital for effective pandemic response and to maintain essential primary health care during such crises [53]. Similar challenges and investments have been noted and called for respectively in high-income settings where volunteers as part of CHSs supported COVID-19 response efforts, like in Canada [56]. This underscores the critical role that CHSs play in supporting the formal health systems, not only in pandemics but also in everyday service delivery efforts [8].

In response, this article builds on existing literature by emphasizing the importance of integrating innovative approaches to address CHW challenges and enhance performance [57]. For example, to address the absence of appropriate technology for health education, health systems should adopt mHealth tools. These tools can support health promotion activities, monitoring, and evaluation [57]. mHealth tools are vital for enhancing the work of CHWs, ensuring professionalism, performance, and scalability of services, especially given pandemic-related mobility restrictions [57].

This study also presents several policy and program implications that could be valuable in similar settings. First, to address ambiguity around CHW roles and access to COVID-19 prevention materials, policymakers should adapt CHW programs to local needs. This requires national consultative CHW policy development processes [58]. Furthermore, evolving governance mechanisms for CHW programs during pandemics is important [58]. For example, policies should prioritize CHWs in receiving the COVID-19 vaccine, and periodically train CHWs in COVID-19 preventive measures [54].

It is important to recognize that the COVID-19 policy landscape was volatile due to the novel, dynamic, and fast-paced nature of the pandemic [59]. Policymakers must strike a balance between responsiveness and consistency. This ensures that communities and support systems like CHWs can function effectively. Although difficult, achieving this balance is crucial to avoid unintended consequences such as demoralizing frontline workers. It also highlights the need for resource investment to maintain this balance [60].

### Strengths and limitations

One main strength of this scoping review lies in the extensive search of the literature on the roles of the CHWs during the COVID-19 pandemic. The inclusion of studies utilizing different methodologies from across the world, including mixed-methods papers and reviews, provided in-depth insights into roles, challenges, and strategies for enhancing the performance of CHWs in the response to COVID-19, and future pandemics in similar settings. One of the limitations, was the possibility of missing out some publications. We tried to mitigate this by conducting several searches and searching the references of publications that we included in the review.

## Conclusion

There is substantial evidence that community health workers (CHWs) played a vital role as trusted community actors during the COVID-19 pandemic. Their engagement was essential in building sustainable and resilient community-based responses to COVID-19 and other infectious diseases. This was especially true in promoting behavioral change at the community level.

Specific roles of CHWs included health promotion and education, surveillance, contact tracing, maintaining essential primary health services, facilitating referrals, advocating for clients and communities, and supporting vaccination efforts.

Despite their significant contributions, CHWs faced numerous challenges that affected their performance. Many experienced stigma and discrimination from community members. Others lacked adequate training in infection prevention and control. They also received insufficient incentives and struggled with shortages of supplies and resources.

Addressing these challenges requires targeted investments to better integrate CHWs into health systems. This will enhance the credibility and sustainability of CHW programs during pandemics.

Adopting innovative approaches, such as mobile health (mHealth) tools, can also support CHW performance and supervision. These tools help CHWs deliver pandemic-related services while maintaining routine primary health care.

It is also crucial to prioritize CHWs for COVID-19 vaccinations. Ongoing training in preventive measures related to the pandemic must be provided as well.

Finally, we recommend that future systematic reviews conduct deeper comparisons across regions to better understand contextual factors essential for the development of strategies aimed at enhancing the performance of community health workers in responding to infectious diseases.

## Electronic supplementary material

Below is the link to the electronic supplementary material.

Supplementary Material 1

## Data Availability

No datasets were generated or analysed during the current study.

## Abbreviations

CHWs: Community health workers
LMIC: Low- and middle-income country
